# Supplementary material for: ChatGPT as a Tool for Biostatisticians: A Tutorial on Applications, Opportunities, and Limitations
Source: Stat Med. 2025 Oct 23;44(23-24):e70263. doi: 10.1002/sim.70263 (PMC12548020; doi:10.1002/sim.70263)
Supplement: Supplementary file 1 — Data S1. Supporting Information. [file SIM-44-0-s001.zip › Supplementary Materials Additional.zip!/3.1_Systematic_Reviews_and_Meta_aspects/3.1_Data Extraction - o4-mini - Chat 4.pdf]

Use Case Meta-Analysis - Data Extraction - o4-mini - Chat 4

Rudloff et al. - 2009 - Concurrent lobular neoplasia increases the risk of ipsilateral breast cancer recurrence in situ treated with breast-conserving therapy.pdf
PDF

Vidali et al. - 2012 - Conservative treatment of breast ductal carcinoma in situ results of an Italian multi-institutional.pdf
PDF

The aim is to perform a meta-analysis on total ipsilateral local recurrence rates at 10 years in cases of ductal carcinoma in situ.

Data inclusion rules are: To ensure the validity of the follow-up data, the results should be as close to 10 years follow-up (minimum) as the information in each study allows. If there is more than one publication from an institution or group, the most recent study with appropriate follow-up will be used to extract 10-year data.

Please provide from each of the two attached studies:

- the rate of ipsilateral local recurrence for conservative surgery with additional radiotherapy (no other interventions or drugs allowed) after 10 years

- the number of eligible patients

- period of data collection

- duration of follow-up

- type of study

- age of patients (mean or median, maximum and minimum)

- treatment modalities

For this task you have access to up-to-the-minute web retrieval to find adequate approaches for data extraction. When answering the task above please proceed as follows:

1. Fetch the most relevant sources.

2. Show your step-by-step reasoning.

3. At the end, give me a concise answer citing the sources you used.

4. Please provide a dataframe with one entry for each study, and column names "study", "rate", "n_patients", "period", "follow_up", "study_type", "age", and "treatment".

Step-by-step reasoning

1. **Identify eligible patient cohorts**
   - **Rudloff et al. (2009)**: 294 women with DCIS treated at a single institution between 1991–1995 with breast-conserving therapy (BCT), defined as conservative surgery + external-beam radiotherapy (no chemotherapy; 6% received tamoxifen).
   - **Vidali et al. (2012)**: 586 women with DCIS treated across 10 Italian centres between February 1985–March 2000, all undergoing conservative surgery + whole-breast radiotherapy (no other adjuvant drugs).
2. **Extract key trial parameters**
   - **Periods of data collection** and **number of eligible patients** are directly reported in the methods of each study.
   - **Duration of follow-up** is given as median follow-up.
   - **Study type** is explicitly described: retrospective cohort (institutional) for Rudloff; retrospective multi-institutional for Vidali.
   - **Age of patients**: median and range are provided.
   - **Treatment modalities**: both studies specify conservative surgery with external beam RT (50 Gy median), with boost in approximately half of Vidali's cohort.
3. **Determine 10-year ipsilateral local recurrence rates**
   - **Rudloff et al.** report 10-year actuarial IBTR = 22% for the overall BCT cohort.
   - **Vidali et al.** report a 10-year risk of local recurrence of 9.6% (Kaplan-Meier estimate).
4. **Assemble concise results table**
   - Create a dataframe with columns: "study", "rate", "n_patients", "period", "follow_up", "study_type", "age", "treatment".

Concise answer

study	rate	n_patients	period	follow_up	study_type	age	
Rudloff et al. 2009	22% at 10 y	294	1991–1995	median 11 y (0–16 y)	Retrospective single-institution cohort	median 55 y (26–89 y)	Br[illegible] su[illegible] ext[illegible] (50[illegible] op[illegible] ch[illegible] tar[illegible]
Vidali et al. 2012	9.6% at 10 y	586	Feb 1985–Mar 2000	median 136 mo (16–292 mo)	Retrospective multi-institutional cohort	median 55 y (29–84 y); 32 ≤ 40 y	Co[illegible] su[illegible] wh[illegible] (m[illegible] bo[illegible] oth[illegible]

- **Rudloff et al.** (n = 294): 10-year actuarial ipsilateral breast tumor recurrence = 22%; conservative surgery + radiotherapy between 1991–1995; median follow-up = 11 years; retrospective single-center; median age 55 years (range 26–89); BCT with external-beam RT (50 Gy, boost optional), no chemotherapy, tamoxifen in 6% .
- **Vidali et al.** (n = 586): 10-year actuarial local recurrence = 9.6%; treated Feb 1985–Mar 2000; median follow-up = 136 months; retrospective multi-institutional; median age 55 years (range 29–84; 32 patients ≤ 40 y); conservative surgery + whole-breast RT (median 50 Gy; boost in 50%); no other interventions .

Quellen
